# A mixed methods study examining the impact of primary health care financing transitions on facility functioning and service delivery in Kenya: a study protocol

**DOI:** 10.12688/wellcomeopenres.21173.1

**Published:** 2024-04-24

**Authors:** Rose Nabi Deborah Karimi Muthuri, Jacinta Nzinga, Benjamin Tsofa, Anita Musiega, Peter Mugo, Ethan Wong, Caitlin Mazzilli, Wangari Ng’ang’a, Brittany Hagedorn, Gillian Turner, Anne Musuva, Nirmala Ravishankar, Felix Munene Murira, Edwine Barasa

**Affiliations:** 1 Health Economics Research Unit, KEMRI Wellcome Trust Research Programme, Nairobi, Kenya; 2 Bill & Melinda Gates Foundation, Seattle, Washington, USA; 3 ThinkWell, Washington, USA; 4 ThinkWell Kenya, Nairobi, Kenya; 5 Center for Tropical Medicine and Global Health, Nuffield Department of Medicine, University of Oxford, Oxford, England, UK

**Keywords:** Primary Health Care, Donor transitions, User fees forgone, Mixed methods, Health financing, Kenya, Study protocol

## Abstract

**Background:**

Kenya has experienced several health financing changes that have implications for financing primary healthcare (PHC). These include transitions from funding by two key donors (the World Bank and the Danish International Development Agency (DANIDA)) and the abolishment of conditional grants that were earmarked for financing primary healthcare facilities. This protocol lays out study plans to evaluate the impact and implementation experience of these financing changes on PHC facility functioning and service delivery in Kenya.

**Methods/design:**

A sequential mixed methods design will be applied to address our research objectives. Firstly, we will perform a document review to understand the evolution of policy changes understudy. Second, we will conduct an interrupted time series analysis across all 47 counties in Kenya to assess these financing changes' impact on health service utilization in all public primary healthcare facilities (level 2 and 3 facilities). Data for this analysis will be obtained from the Kenya Health Information System (KHIS). Third, we will carry out in-depth interviews with health financing stakeholders at the national, county, and health facility levels to examine their perceptions of the experiences with these changes in health financing.

**Discussion:**

This mixed methods study will contribute to evidence on the sustainability of financing primary healthcare in low and middle-income countries facing financing changes and donor transitions.

## Plain language summary

Evaluating the Impact of Primary Healthcare Financing Transitions on PHC Facilities in Kenya.

In 2020, funding allocated for public primary healthcare (PHC) facilities was eliminated as conditional grants in Kenya. Through the support of the PHC-specific conditional grants, public PHC facilities would provide free healthcare services to patients.

Additionally, the World Bank and Danish International Development Agency (DANIDA) are transitioning from providing funding support to PHC facilities in Kenya. DANIDA's PHC support grant will be terminated at a 25% yearly rate over four years, coinciding with the end of the World Bank Transforming Health Systems programme for Universal Health Care. Before obtaining the financing, these grants had county-specific requirements known as service performance objectives.

These financing changes will likely impact the level of financing that PHC health facilities will access. Hence, the proposed study examines the impact of these financing changes on PHC facilities functioning and service delivery in Kenya, as well as the implementation experience of stakeholders in the health sector.

## Background

Kenya has committed to achieving Universal Health Coverage (UHC) through strengthening Primary Health Care (PHC) by 2030 ( Government of Kenya, 2020b). UHC refers to all individuals having access to the complete spectrum of high-quality health services as needed without suffering financial hardship ( World Health Organization, 2010). PHC is the initial point of care that people and families may access inside their community (in Kenya, for example, PHC consists of level 1 care provided by community health workers, as well as level 2 and 3 healthcare institutions), with full involvement from the participants, and at a cost that the nation and community can afford ( Bryant & Richmond, 2017).

Attaining good population health at a low cost entails actively and consistently investing in PHC ( Balabanova *et al.*, 2013). The financing of PHC determines health system performance. PHC financing involves investing, incentivizing, and optimizing resource allocation to facilitate PHC provision based on a country's socioeconomic and political factors ( Goodyear-Smith *et al.*, 2019). Increased investment and efficient spending are positively associated with better performance at the PHC level ( Hanson *et al.*, 2022).

Following the promulgation of the 2010 constitution, Kenya transitioned into a devolved system of government with a national government and 47 semi-autonomous county governments ( Government of Kenya, 2010). In the health sector, the role of the national government is policy formulation and regulation, plus providing tertiary healthcare services. Meanwhile, county governments provide secondary and primary healthcare services. Kenya has a four-tier system of healthcare. Tier 1 is community care (also known as level 1), including community health workers; tier 2 is primary health care (PHC) (comprised of health dispensaries at level 2 and health centres at level 3); tier 3 is secondary care (consist of level 4 and 5 facilities namely sub-county and county hospitals); and at tier 4 is the tertiary care (consisting of the national level referral hospitals and institutions at level 6) ( Ministry of Medical Services and Ministry of Public Health and Sanitation, 2009).

The sources of funding for the Kenyan health system include 1) government transfers, 2) out-of-pocket spending, 3) external aid, 4) voluntary health insurance contributions, and 5) social health insurance contributions ( World Health Organization, 2020). The sources of funds for PHC facilities include the national treasury, the county government, the National Health Insurance Fund (NHIF), and donors ( Kairu *et al.*, 2021). Due to lost revenue from user fees, the GOK 2013 introduced a conditional grant known as user fees forgone, which involved the national government reimbursing PHC facilities through the county governments.

Two significant PHC financing changes are occurring in Kenya: 1) the transition of donors that fund PHC and 2) abolishing the user fees forgone (UFF) conditional grant in 2020. Donor transitions entail officially transferring donor financing and other responsibilities for health programs to one or more implementing partner(s), such as the government, to promote the program's sustainability ( Bennett *et al.*, 2015; Gotsadze *et al.*, 2019). Two major donors who fund PHC in Kenya are transitioning: the World Bank Transforming Health Systems (WB THS) for UHC and the Danish International Development Agency (DANIDA). Between June 2016 and September 2023, the World Bank THS funded improvements towards reproductive, maternal, newborn, child, and adolescent health services ( World Bank Group, 2016).

DANIDA is a bilateral donor that agreed to support Kenya's PHC strengthening program between 01 January 2021 and 31 December 2025 ( Office of the Auditor-General, 2022). They aim to promote RNMCAH by supporting outreach programs, operational functions, and maintenance costs within PHC facilities ( Office of the Auditor-General, 2022). However, DANIDA began phasing out in 2022, which is the year the implementation of the counterpart funding modality began, to phase out entirely by 2025 ( Denmark Ministry of Foreign Affairs, 2021).

In 2020, the national GOK abolished the funds earmarked for user fee forgone conditional grants to the non-conditional county government equitable share allocation. Counties no longer receive the user fee forgone conditional grant from the national government. A conditional grant is an intragovernmental grant from the national government to the devolved governments with particular conditions or standards ( Chen *et al.*, 2014). In this case, the conditions were that the UFF conditional grant allocation from the national government is allocated only to PHC facilities. Conditional grants can be used as 1) an incentive, e.g., in this case, for counties to prioritize PHC service provision; 2) a financial tool that facilitates accountability; 3) a rationing decision-making tool where the national government guides a newly devolved government on what and how to prioritize specific agenda ( Bischoff & Blaeschke, 2010; Chen *et al.*, 2014)—introducing the user fee forgone (UFF) conditional grant in 2013 restored PHC facilities' autonomy in terms of health budgeting, planning, and spending. Intuitively, the loss of funding means resource reduction at the PHC level. The funds from the UFF and the donor grants were often used for operations and to plug supply and HRH gaps. Hence, we plan on exploring what this resource loss will mean for PHC facility functioning and overall service delivery.

Bridging the existing research gap on the impact and experience of these PHC financing transitions is paramount for evidence-based decision-making. Thus, the proposed study will investigate the impact and experience of PHC financing donor transitions and GOK abolishing UFF conditional grants on PHC performance and service delivery.

## Overarching objective

The overarching objective of this study is to examine the experience and impact of donor transitions and the abolishment of the user fees forgone conditional grant on PHC health facility performance and service delivery in Kenya. The specific objectives of this study are:

1. To assess the impact of donor transitions and the abolishment of the user fees forgone conditional grant on PHC facility functioning and service delivery in Kenya.

2. To assess the implementation experience and gendered effects of donor transitions and abolishing the user fee forgone conditional grant in Kenya.

## Study justification

Public PHC facilities in Kenya budgets were funded substantially by donor conditional grants and the UFF conditional grant. Recently, two major donor funders, World Bank THS and DANIDA, are phasing out. Both donors were committed to developing and strengthening the PHC system by funding the facilities to reach the frontline. World Bank THS focused on PHC programs such as maternal, reproductive, child and adolescent health care programs. At the same time, DANIDA funded the operations of PHC facilities majorly. PHC facilities have, therefore, lost three significant sources of PHC funding.

Further, these conditional grants were accompanied by accountability and governance arrangements, likely to be compromised by this change. Donor funds and UFF provided: 1) direct funding to the frontline, guaranteeing autonomy 2) Additional accountability criteria, including conditions, audits, etc.; and 3) system support overall, all of which may be jeopardized by the withdrawal of UFF PHC conditional grant and the transfer of donor funds. Conditional grants also require that PHC facilities have some autonomy over their use. It is not known what effect these transitions will have on health facility autonomy.

Research shows that gender is a crucial component that influences and predetermines health outcomes and systems ( Hay *et al.*, 2019). Among the gaps in knowledge this study aims to bridge are the gendered implications of PHC financing transitions from abolishing the UFF, WB, and DANIDA donor transitions, which we shall investigate in this study. In 2006, a UN resolution urging countries to mainstream gender into public policy and programs was adopted ( Secretary-General, 2010). In 2015, among the 17 Sustainable Development Goals (SDGs), gender equality was the fifth goal, and Kenya is among the nations committed to working towards gender equality (SDG number 5).

Hay *et al.*, 2019 reported that gender equality policies that go beyond numeric distributions, for instance, higher percentages of female physicians, are associated with better health outcomes; nevertheless, gender parity alone is not enough to attain gender equality—creating an enabling environment that develops and implements gender policy results in positive outcomes. For instance, institutional respect and support for nurses enhance treatment quality, improve responsiveness within the health system, and improve health outcomes ( Hay *et al.*, 2019). *Gender* is a social construct that stems from biological differences between the sexes. Male and female interactions differ following their interaction with the health system as professionals, patients, and policymakers. Hence, we aim to explore the differences in implementation experiences of PHC financing abolishment and transitions that could differ between males and females, potentially directly or indirectly influencing the nuances within the PHC system.

## Methods

### Conceptual framework

**Figure 1.**
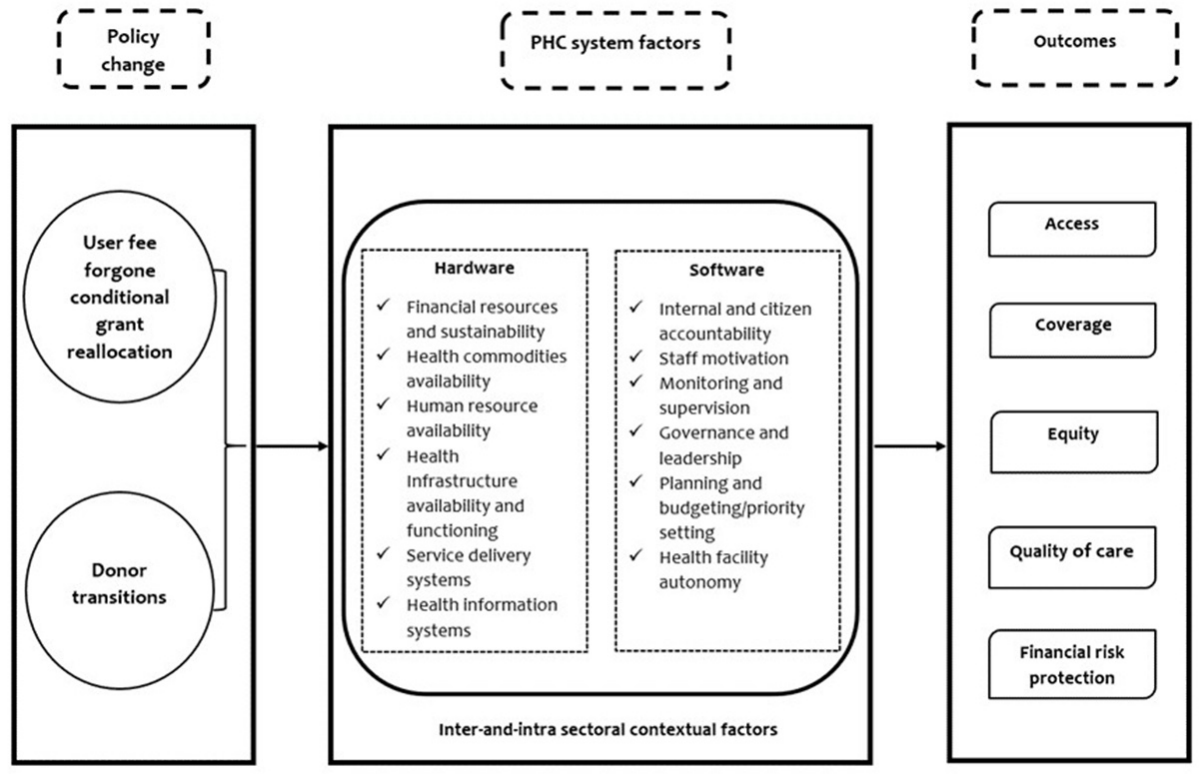
Study conceptual framework. Source: Authors' own.

The conceptual framework presented in Figure 1 illustrates how two PHC financing policy changes PHC system factors and other sectoral context factors to influence outcomes related to PHC financing, service delivery, and performance in Kenya.

The framework assumes that the two financing transitions will likely influence PHC systems factors categorized into hardware and software components. In this framework, the hardware factors include financial resources and sustainability, health commodities availability, human resource availability, health infrastructure availability and functioning, and service delivery systems. The software components in this study include internal and citizen accountability, staff motivation, monitoring and supervision, governance and leadership, planning and budgeting/priority setting, and health facility autonomy; how the influence of these system factors is impacting health system goals that include access, coverage, equity, quality of care, and financial risk protection. The framework also considers the influence of inter-and-intra contextual factors such as political will, commitment, community involvement and engagement, economic status, and other possible sectors, which have overt or covert effects on the PHC financing transitioning process we focus on in our study.

### Policy change

On 01 June 2013, the president announced the ban on the PHC facilities' user fees. Before this announcement, patients visiting PHC facilities paid KES 10 for dispensaries and KES 20 for health centres. The new reform immediately increased the demand for services at PHC facilities. However, county governments had yet to budget for this in FY 2012/13. To support the loss of revenue from abolishing user fees in health dispensaries and health centres, the national GOK introduced the UFF conditional grant. All county governments would receive a conditional grant to spend on only PHC services at the level 2 and level 3 health facilities, implemented from FY2014/15 to FY 2020/21. For example, in FY 2020/21, the total allocation of the UFF conditional grant was KES 900,000,000. The allocation criteria of the UFF conditional grant entails distributing funds according to the population data and outpatient attendance (OPD) workload as reported in the DHIS, now KHIS ( Government of Kenya, 2020a).

In 2020, because of contestations and stalemate that affected the development of the national budget and the division of revenue bill (which allocates resources between national and county government) that year, the president offered to break the stalemate by freeing more funds that were under the control of the national government, to be placed in the non-conditional equitable share for county governments. In doing this, the president offered conditional funds that had been earmarked for the user fee forgone by the national government, which would be given to counties as part of the consolidated equitable share. This political decision broke the deadlock that had ensued, leading to PHC facilities losing these funds.

A second policy change we plan to explore is the impact of PHC financing donor transitions. Two major donors are phasing out/transitioning, specifically the World Bank (WB) and DANIDA. The World Bank THS project allocated funds totalling an estimated KES4.3 billion ( Government of Kenya, 2020a). This project aimed to improve the delivery, utilization, and quality of PHC services focused on reproductive, maternal, newborn, child, and adolescent health (RMNCAH) at the county level ( World Bank Group, 2016). According to the financial agreement between the World Bank and the GOK, the World Bank THS project's allocation criteria were 20% of each year's annual allocation according to the revenue allocation formula and 80% allocation according to the performance formula. Allocation adjustments are made according to how well a county transfers funds the year before ( Government of Kenya, 2020a).

DANIDA is financing Kenya's UHC devolved system program ( Denmark Ministry of Foreign Affairs, 2021; Government of Kenya, 2020a). The DANIDA grant aims to improve the access and quality of PHC and RMNCAH services at the county level ( Denmark Ministry of Foreign Affairs, 2021). According to the financing agreement between the GOK and DANIDA, the allocation criteria for disbursing funds to the counties is according to the County Revenue Allocation ratio (CRA) ( Government of Kenya, 2020a). The DANIDA grant for UHC to Kenya is subject to several requirements such as counties shall distribute the grants following clear criteria shared with the Project Management Team; funding is only for health dispensaries and centres; after one year, at least 20% of the county budget exempt of conditional grants must be to health; reimbursing county governments promptly from the SPA, and the county governments reimbursing the health facilities via the IFMIS is paramount ( Government of Kenya, 2020a). Since the DANIDA funds went directly to facilities, while most of the THS funds were used at county level. We shall assess the impact of withdrawal of these funds from that lens as well.

The process of the PHC financing transitions varies with the donors. For instance, DANIDA uses a matching financing strategy that aims for a 25% decrease per fiscal year (FY) starting in 2020/21 for three years. During the first year of the PHC financial transition by DANIDA, for example, they covered 75% of the expenditures associated with PHC, and the counties covered 25% of those costs; in the second year, DANIDA covered 50% while the counties covered 50% and so on until the counties will cover 100% of the costs ( Government of Kenya, 2021). For instance, the DANIDA funding of KES 900 million decreased to KES 701 million between FY 2020/21 and 2021/22 ( Government of Kenya, 2021). Under the World Bank THS for the UHC grant, which is also in transition, financing was withdrawn in FY 2022/23, after the initial reduction from 4.3 billion in FY 2020/21 to 2.2billion in FY 2021/22 ( Government of Kenya, 2021).

### Study setting

We will conduct this study in Kenya, a lower-middle-income country in the East of Sub-Saharan Africa. Nationally, we shall collect quantitative data and perform a document review. We will focus on counties representing geographical and economic differences at the county level. Table 1 shows county characteristics that show the variation in the selection of the counties by geographical variation and socioeconomic factors such as GDP and population size. For instance, it is evident that the life expectancy of both males and females, health facility births, gross domestic product (GDP), number of health facilities, and NHIF coverage are all highest in the urban county of Nakuru with the highest GDP, followed by the rural county of Kakamega with the second highest GDP, and lastly, Isiolo county which is remote with the lowest GDP. These attributes have potential impacts on the outcomes that could be attributable to multiple factors, including resource availability.

**Table 1.** County characteristics.

Characteristics	Nakuru County *	Kakamega County **	Isiolo County ***
Population **	1.8 (million)	2.6 (million)	185.9 (thousand)
**Geographical criteria**	Urban	Rural	Remote
**% Under Age 5**	16.19%	17.49%	14.3%
**% Females**	49.82%	51.76%	48.57%
**Gross domestic product (GDP) (Millions in KES)**	517,462	182,563	15,850
**Male life expectancy at birth**	67.6 years	64.6 years	59.7 years
**Female life expectancy at birth**	71.7 years	69.3 years	66 years
**Fertility rate**	3.4	3.4	3.5
**Health facility births (%)**	69.7%	47%	42.1%
**Contraceptive prevalence**	53.5%	60.3%	26.3%
**Total number of health facilities**	424	272	54
**Total number of health dispensaries (level 2)**	163	122	37
**Total number of health centres (level 3)**	31	55	3
**NHIF coverage**	34.2%	30.2%	20%
**Total government health spending (per capita, KES)**	1,725	1,277	3,670

*( **Ministry of Health, 2015c**)

**( **Ministry of Health, 2015b**)

***( **Ministry of Health, 2015a**)

### Study design

We will use a sequential mixed methods study design. The document review will come first and will eventually be used in conjunction with the interrupted time series analysis (ITSA) and in-depth interviews. After the policy changes we are evaluating on PHC funding transitions, quantitative research will be conducted utilizing an ITSA design to determine trends in study outcomes. The third stage, the qualitative component, will explore the gendered impact and implementation experience of the GOK's withdrawal of the UFF PHC conditional grant and the DANIDA and WB's PHC funding changes.

### Quantitative study

**Figure 2.**
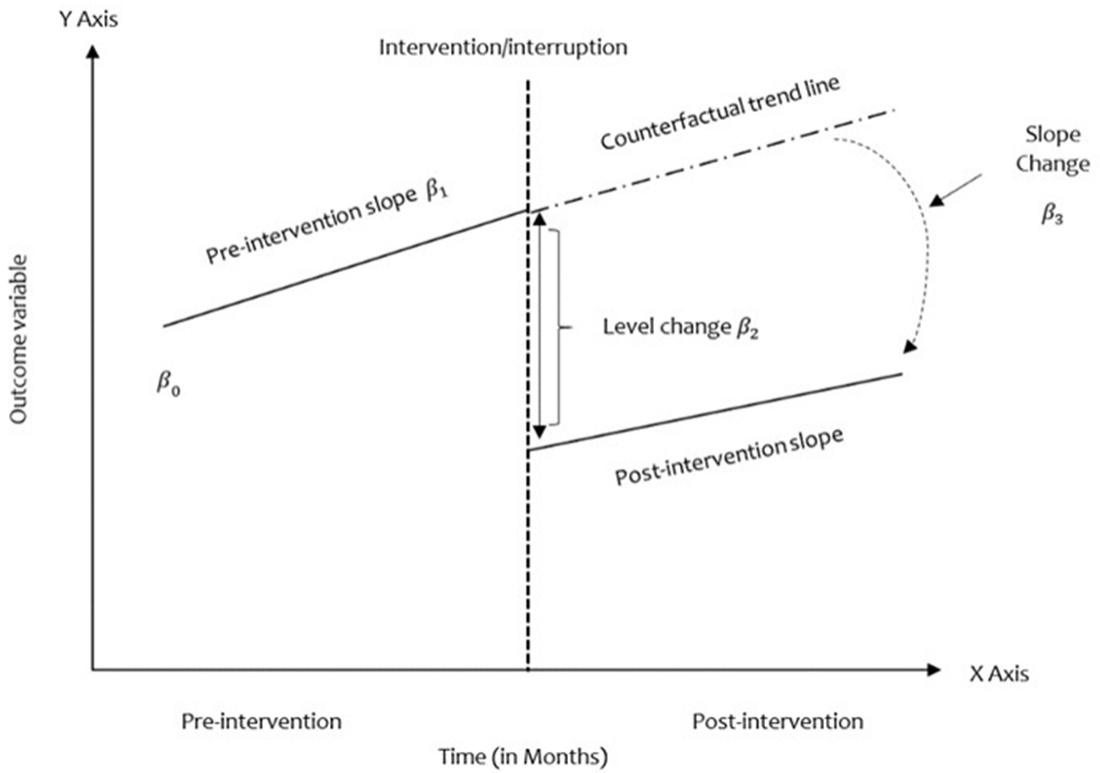
ITSA impact model. Source: Authors' own.

***Study design.*** Using a quasi-experimental, longitudinal approach, we will apply an ITSA design to answer our first objective. An ITSA is a functional research design for assessing the efficacy of population-level health interventions ( Bernal *et al.*, 2017). We will use the ITS to measure the changes in levels and trends in our outcomes of interest due to the policy changes, namely GOK abolishing the UFF and the PHC financing transitions by WB and DANIDA. We will conduct the ITS analysis step-by-step. Firstly, we shall maintain clear differentiation by having precise pre-and-post-intervention timings. We will collect data before and after the policy changes, constituting interruptions in different financial years (FY) (as shown in Figure 2). Interruptions in this study will be the points at which the funding was cut off or reduced. The data points will be monthly, showing the trends of the outcomes.

Based on our ITS design, we will capture data on our outcomes of interest at several evenly spaced time points, in our case, capturing monthly trends (i.e., 12 months before the interruption and 12 months after). While the abolishment of the UFF by the GOK took place in one interruption, the PHC financing transitions by WB and DANIDA were/are phased. We will evaluate step and trend changes in the outcome variables across the varied periods; these are a) Abolishment of GOK UFF PHC conditional grant with one interruption time point: FY 2020/21; b) The World Bank THS-UCP grant with two interruption points: FY 2021/22 & 2022/23; and c) The DANIDA PHC support grant with four interruption points (4): FY 2020/21, 2021/22, 2022/23, 2023/24.

Secondly, we will conduct the analysis in two-fold descriptive and regression analyses (described in detail in subsequent sections on data analysis). Third, we look into the ITS methodological issues that may arise, such as the effects of seasonality that contribute to autocorrection. We will use either one of the following methods to solve the problems related to autocorrelation: the autoregressive intreated moving average (ARIMA) or prais regression ( Bernal *et al.*, 2017). Finally, we will perform sensitivity analyses and model-checking by plotting residuals and partial correction to assess the effect of time lags and to adjust for seasonality.

***Study population.*** Kenya's 47 counties will comprise the national sample population for the quantitative study's ITS analysis (ITSA). We will exclusively concentrate on evaluating the ITS trends across PHC facilities, namely in level 2 (health dispensaries) and level 3 (health centres). The PHC outcome variables we will explore for the ITSA in Table 2 illustrate the RNMCAH projects that DANIDA and WB intended to support with their contributions to fortify the PHC system.

**Table 2.** Outcome variable list and descriptions.

Indicator name	Indicators description	KHIS Description	Source	Availability
Utilization	Number of outpatient visits per level 2 and 3 facilities and by county	OPD visits	KHIS	Monthly for level 2 and 3 facilities
Reproductive/maternal	Number of Women (aged between 15–49) that had 4 or more antenatal visits during last pregnancy	4 ANCs visits	KHIS	Monthly for level 2 and 3 facilities
Reproductive/maternal	Number of deliveries conducted by skilled birth attendants	Deliveries	KHIS	Monthly for level 2 and 3 facilities
Childhood immunization	Number of children under who have received 2 doses of the vaccine against Measles and Rubella	Measles and Rubella	KHIS	Monthly for level 2 and 3 facilities
Childhood immunization	Number of children under 1 year receiving DPT3 vaccine	DPT3	KHIS	Monthly for level 2 and 3 facilities

***Data collection.*** Secondary data will be extracted from the following sources: Kenya Health Information Systems (KHIS). Table 2 includes the outcome variable names, descriptions, data sources, data collection method and periods available with data for the quantitative study across the 47 counties.

***Data analysis.*** Firstly, we will perform a descriptive analysis of the outcome variables and present the results in scatter plots and summary statistics in tabulated form. The scatter plots will capture the underlying trends, seasonal patterns, and outliers ( Bernal *et al.*, 2017). Secondly, we will conduct a regression analysis guided by the model and pictorial (see Figure 2) below to show the associations between variables and period for the ITS, driven by the standard model below ( Bernal *et al.*, 2017):


$$Y_{t}\;=\;\beta_{0}\;+\;\beta_{1}T\;+\;\beta_{2}X_{t}\;+\;\beta_{3}TX_{t}\;(1)$$

Where in equation (1):

*Y _t_*: outcome variable at time T

T: time-lapse from the beginning of the study frequency (Unit: month or year)

*X _t_*: dummy variable pre-intervention (0) or post-intervention (1)

*β*_0_: baseline level at T=0

*β*_1_: the change in outcome associated with a time unit increase (pre-intervention trend)

*β*_2_: level change following the intervention

*β*_3_:the slope change following the intervention (Time vs Intervention *TX _t_*)

An ITS's primary goal is to determine whether the post-intervention data pattern differs from the pre-intervention pattern ( Hudson *et al.*, 2019). For instance, a change in level or slope relates to the difference between the time point of interest and the expected pre-intervention trend or the difference between the post- and pre-intervention slopes, respectively (see Figure 2). Various effect estimates will be available to quantify the effect of interventions/interruptions.

### Qualitative study

***Study design.*** The qualitative research aspect will entail a document review and in-depth interviews. The document review will entail document analysis, capturing details on the policies under the existing documents related to abolishing the UFF conditional grant and donor transitions. To complement the findings from the document review, to gain an in-depth understanding of the implementation experience and gendered effects of abolishing the UFF conditional grant and the WB and DANIDA WB PHC financing transitions. The cross-sectional qualitative interview design means we will be interviewing participants at a single point in time.

The information retrieved from the document review will mainly describe the genesis of the UFF and the PHC financing grants from DANIDA and WB, which is the 'what'. From the document review, we will explore critical outcome variables in the ITSA. The in-depth interviews will then delve into understanding the trends in the outcomes of interest captured in Table 2 and based on gaps in the document review by capturing the 'how?' and 'why?' -will entail explanations and reasoning behind the causal relationships from the quantitative research and document analysis.

***Sample population.*** Participants in the health system from both the national and subnational levels will make up the study population. To participate in the study, we will extend invitations to national-level officials holding different MOH and donor roles who assisted in the PHC finance transition process. Those in various PHC-related roles, including directors, PHC facility managers, and county health management team members, will be invited to participate in this study as county-level participants.

***Sample size and sampling.*** We will collect data from the national level and three purposively selected counties. At national and county levels, we will select stakeholders involved in health financing and PHC service delivery decision-making and implementation. At the county level, we aim to interview 5–8 key informants holding the following positions: County Director of Health, County Executive Committee Member of Health, Chief Officer of Public Health, Chief Officer of Medical Services, a CHMT member, Director of Administration and Planning/Primary Health Care Coordinator, Level 2 Health Facility Manager, Level 3 Health Facility Manager. At the national level, our potential participants will be those occupying the following positions: Head of the Department of Primary Health Care, Division of Healthcare Financing, Division of Healthcare Quality, Member of the National Treasury/Council of Governors Member of Health Financing, a DANIDA representative and, a World Bank THS-UCP representative. Through purposive sampling, participants will select those who have in-depth knowledge of the study subject because of their roles and or experience of the phenomenon of the study. At the point of saturation, we shall stop interviewing more participants. The interviews at the national level will stop once we hit the point of saturation ( Saunders *et al.*, 2018).

***Data collection.*** We will collect qualitative information using a combination of semi-structured in-depth interviews and document review. The document review will complement the qualitative and quantitative aspects of the study. The administrative documents and relevant papers that contain information on the UFF conditional grant and donor transitions by DANIDA and WB will be examined including the following (based on availability): Kenya's Primary Health Care Strategic Framework 2019–2024, Kenya National Health Accounts from the years available e.g., 2001, 2003, 2005/6, 2015/16 & 2019, Kenya Primary Health Care Network Guidelines, Primary Health Care Systems (PRIMASYS) case study, World Bank THS reports, DANIDA reports, peer-reviewed articles on PHC financing transition reforms in Kenya, ThinkWell reports on PHC in Kenya. The document review will collect information on the following: author(s), year, objective, specific PHC financing transition, what was financed by the grant, how it was financed, outcomes/goal performance of the grant, and challenges.

The in-depth interview topics include general PHC financing sources, questions examining the relationship between UFF abolishment and PHC financing donor transitions, experience with health infrastructure, service delivery systems and facility functionality, health information systems, accountability mechanisms, governance and leadership, autonomy of health facilities, and the relationship between all these PHC system factors with outcomes in the context of Kenya will be covered by the qualitative interview guide.

***Data analysis.*** The interviews will be transcribed using Microsoft Word. To maintain the anonymity of the participants, we will assign each interviewer a code. We shall provide codes such as II1, II2, or II3 etc for the transcripts and only provide general role descriptions as a policymaker, development partner, health manager, or health worker).

Using a framework analysis, we shall examine the implementation experience of WB and DANIDA in abolishing the UFF and donor transitions. We shall apply a five-step framework analysis approach, initially developed by Ritchie and Spencer in 1994 ( Srivastava & Thomson, 2009).

The steps are as follows: 1) we shall familiarize ourselves with the transcripts by listening to audiotapes and reading the transcripts and field notes to capture the key emerging themes and concepts, some of which may be recurrent; 2) using a thematic framework guided by the conceptual framework presented earlier, we will sort and sift through the data based on the priority issues related to our topic of study on the abolishment of the UFF conditional grant and donor transitions by DANIDA and WB. The second step will involve refining the thematic framework from a logical and intuitive thinking process. 3) Third indexing will be done by applying a numeric system for the index reference merged with a primarily descriptive process rooted in abolishment of the UFF conditional grant and donor transitions at the PHC level in Kenya; 4) charting will be the fourth step comprised of arranging the specific pieces of data according to a thematic chart with the core themes, which will be in an ideal format for presenting the research findings; 5) mapping and interpretation will be the final stage encompassing the research team pulling together the significant characteristics through a process that will entail "defining concepts, mapping range and nature of phenomena, creating typologies, finding, associations, providing explanations, and developing strategies." P.186 ( Ritchie & Spencer, 2002). Finally, we shall synthesize the emerging findings.

### Validity and reliability of the study

In our quantitative study, we will measure content and construct validity. We will access content validity via face validity. Face validity will be measured by engaging stakeholders and experts via a workshop to review and provide their opinions on the variables and sources of the secondary data we use to measure the understudy concepts. We will measure construct validity specifically through a theory/literature evidence approach. Whether abolishment of the UFF conditional grant and WB and DANIDA PHC financing transitions) will be a gradual change in the gradient of the trend, a change in the level, or both, and whether the change will follow the intervention immediately or whether there will be a lag period before any effect, we will hypothesize how the intervention would impact the outcome if it were practical. We will develop impact models for each outcome variable based on existing evidence. The impact models must be made a priori in light of the available research, knowledge of the intervention, and understanding how it may influence the outcome ( Bernal *et al.*, 2017). Developing impact models on outcome data increases the possibility that an effect may be found owing to random fluctuations, leading to irreducibly complex conclusions about the intervention's effectiveness ( Bernal *et al.*, 2017). Therefore, we will base the impact models on existing literature rather than the outcome data to avoid bias compromising validity.

In this study, we shall ensure trustworthiness and rigour through several strategies: triangulation, credibility, transferability, dependability, and thick description. We will apply data triangulation by merging the findings from the in-depth interviews with those from the document review. We aim to ensure the credibility of our findings through member-checking the interviews following transcription and initial analysis. By providing in-depth information on the context and methods used in the interviews, we aim to ensure the transferability of the findings. Dependability is "the trust in trustworthiness", which is about ensuring data stability over time and conditions ( Stahl & King, 2020) through audit trailing consisting of process logs of all the activities the research team will do, including decisions made during the study to ensure dependability. We will use thick descriptions to provide readers with writings full of specifics, capturing the nuances in the actions or reforms described to the extent that they are palpable ( Stahl & King, 2020). Providing adequate contextual knowledge through thick description will also be accompanied by document reviews that the research team will have selected in consultation with various stakeholders.

### Data management

The document review data extracted from relevant publications and administrative records will be entered into an Excel spreadsheet, stored in password-protected servers, and accessible to the research team. The qualitative data will be stored securely on safe, password-protected servers. The recordings will be in a shareable file accessible to only the research team. Since the quantitative research component is secondary data from KHIS, it will not include personally identifying information. At the same time, the qualitative data in terms of the recordings will also be stored in the password-protected server and will be accessible only to the research team. After the transcription and initial analysis of the audio recordings, we will delete them, and the transcripts will be kept for analysis for a more extended period following verification of recordings and transcripts within the team.

### Ethical considerations

Ethics approval for this protocol has been obtained from the Scientific & Ethics Review Unit (SERU) at the Kenya Medical Research Institute (KEMRI) Wellcome Trust (KWTRP) in Kenya (protocol number: KEMRI/SERU/CGMR-C/294/4708; date of approval: 03 May 2023). The national research permit from the National Commission for Science, Technology and Innovation (NACOSTI) has also been obtained (permit number: NACOSTI/P/23/28111; date of approval: 28 July 2023). Permission to conduct this study will also be sought from the Council of Governors in the respective counties understudy will also be sought before the commencement of the empirical aspects of this study. Using an informed consent form, we will seek written consent from all participants, The informed consent form has been approved by the Scientific & Ethics Review Unit (SERU). As a requirement of SERU, the researchers have successfully taken a course on Research Ethics Evaluation, which includes understanding and ensuring that our study abides by ethical research principles, including the ones of the Declaration of Helsinki principles, among others.

## Discussion

We plan on using a mixed methods approach that contributes to the understanding, planning, and process of acting on PHC financing transition reforms that are occurring at present and in the future. Through this study, we aim to provide policymakers and implementers with information that could influence their evidence-based plans on PHC financing transition arrangements following the transitions at the county and national levels.

This study will contribute to the body of knowledge on the sustainability of PHC financing in LMICs in a context where there is donor transition and other financing changes. The findings from the study will provide evidence on how low- and middle-income countries experience financing transitions and the implications of financing PHC. This study will also shed light on the gendered effects of the PHC financing transitions by assessing gender disparities in health and health systems that impact PHC facility functioning and service delivery.

This study's potential limitations and challenges are present in the quantitative and qualitative research components. The quantitative analysis may be affected by data availability and quality challenges. Data on all the variables of interest may not be available in the desired temporal scale (monthly). Data quality is likely to be affected by missingness and invalid data entries. To mitigate this, we will assess data availability for all indicators of interest and select variables where data is available in the desired temporal scale. We will use multiple imputations to address missingness. We will apply range checks to check for the validity of the data and replace data that are outside of logical ranges using variable medians. The qualitative component will likely have limitations such as recall and social desirability biases. The recall bias from the interviews will be tackled by conducting a document review of the policy changes under study. On the other hand, reducing social desirability bias will be done by explaining the study's aims, building rapport with the participants, and triangulating the results with the document review.

## Dissemination plan

Publications in peer-reviewed journals and conferences are how we will reach the global scientific community. A summary will be made available to the public and policymakers through blogs, policy briefs, and technical briefs.

All research participants at the national, county, and healthcare institution levels will get summary findings sheets via email, including recommendations for potential actions to enhance the PHC financing transitions of county health systems.

## Ethics and consent

Ethics approval for this protocol has been obtained from the Scientific & Ethics Review Unit (SERU) at the Kenya Medical Research Institute (KEMRI) Wellcome Trust (KWTRP) in Kenya (protocol number: KEMRI/SERU/CGMR-C/294/4708; date of approval: 03 May 2023). The national research permit from the National Commission for Science, Technology and Innovation (NACOSTI) has also been obtained (permit number: NACOSTI/P/23/28111; date of approval: 28 July 2023). Permission to conduct this study will also be sought from the Council of Governors in the respective counties understudy will also be sought before the commencement of the empirical aspects of this study. Using an informed consent form, we will seek written consent from all participants, The informed consent form has been approved by the Scientific & Ethics Review Unit (SERU). As a requirement of SERU, the researchers have successfully taken a course on Research Ethics Evaluation, which includes understanding and ensuring that our study abides by ethical research principles, including the ones of the Declaration of Helsinki principles, among others.

## Data Availability

No data are associated with this article.
