# Supplementary material for: A mixed methods study examining the impact of primary health care financing transitions on facility functioning and service delivery in Kenya: a study protocol
Source: Wellcome Open Res. 2024 Apr 24;9:220. [Version 1] doi: 10.12688/wellcomeopenres.21173.1 (PMC11399766; doi:10.12688/wellcomeopenres.21173.1)
Supplement: Peer review history

# Reviewer report: Reviewer response for version 1

**DOI:** 10.21956/wellcomeopenres.23418.r84274

**Published:** 2024-09-13

**Authors:** Maninder Pal Singh

**Affiliations:** 1 London School of Hygiene and Tropical Medicine, London, UK

The article highlights an important issue that pertains to health financing of many low-and-middle-income (LMICs) countries, especially in Africa where different countries are transitioning from donor-based funding. This shift is to sustainable financing to meet the communities' healthcare needs which are decided by the population and not based on vertical programs.

 It will be an interesting study as it tries to assess the impact of such policy change in Kenya which is a diverse country. The article is well written and methods are sufficiently explained. This protocol and the findings of the study will provide evidence not only for Kenya but for other LMICs.

Is the study design appropriate for the research question?

Yes

Is the rationale for, and objectives of, the study clearly described?

Yes

Are sufficient details of the methods provided to allow replication by others?

Yes

Are the datasets clearly presented in a useable and accessible format?

Not applicable

Reviewer Expertise:

Health Economics of Tuberculosis in LMICs

I confirm that I have read this submission and believe that I have an appropriate level of expertise to confirm that it is of an acceptable scientific standard.

# Reviewer report: Reviewer response for version 1

**DOI:** 10.21956/wellcomeopenres.23418.r89955

**Published:** 2024-08-22

**Authors:** Blake Angell

**Affiliations:** 1 The George Institute for Global Health, University of New South Wales, Sydney, New South Wales, Australia

Thanks for the chance to review this protocol. The authors present their plans to evaluate both the impact and the implementation of Kenya’s transition to new ways of primary healthcare financing. The work proposed examines an important area for healthcare policymaking and investment in Kenya, filling a local gap in published research, that is also likely to be of interest to those working in other nations too. My only suggestions for improvement relate to the description of the methods to be used, particularly for the quantitative study. It is not clear to me how many data points are being included (yearly or monthly) for each variable? Or how the different ‘interruption’ points are being included, it seems the authors plan to try and estimate the impact of each policy change separately? How are the other changes (or interruptions) being controlled for each of these analyses? I think the authors could boost the level of detail of what is planned to be done and this would improve the paper substantially.

Is the study design appropriate for the research question?

Yes

Is the rationale for, and objectives of, the study clearly described?

Yes

Are sufficient details of the methods provided to allow replication by others?

No

Are the datasets clearly presented in a useable and accessible format?

Not applicable

Reviewer Expertise:

Health economics

I confirm that I have read this submission and believe that I have an appropriate level of expertise to confirm that it is of an acceptable scientific standard, however I have significant reservations, as outlined above.

# Reviewer report: Reviewer response for version 1

**DOI:** 10.21956/wellcomeopenres.23418.r87230

**Published:** 2024-07-12

**Authors:** Pr Nasreddine Aissaoui

**Affiliations:** 1 University of Oum El Bouaghi, Oum El Bouaghi, Algeria

Unfortunately, several pieces of information are missing so that the analysis is more fluctuating, among them:

- We cannot find well-detailed historical framework for the development of primary health care in healthcare provision in Kenya.
- We cannot find a figure that explains the pyramidal provision of health care in Kenya; from primary care to specialized and highly specialized care.
- We do not have a clear idea of ​​the characteristics and specificities of healthcare provision in Kenya, in terms of healthcare structures and human resources.
- We do not have a clear idea of ​​the financial, organizational and geographic/demographic barriers that hinder good and equitable health coverage for the Kenyan population.

Is the study design appropriate for the research question?

Partly

Is the rationale for, and objectives of, the study clearly described?

Yes

Are sufficient details of the methods provided to allow replication by others?

Yes

Are the datasets clearly presented in a useable and accessible format?

Yes

Reviewer Expertise:

NA

I confirm that I have read this submission and believe that I have an appropriate level of expertise to confirm that it is of an acceptable scientific standard, however I have significant reservations, as outlined above.
